# Application of Biosensors, Sensors, and Tags in Intelligent Packaging Used for Food Products—A Review

**DOI:** 10.3390/s22249956

**Published:** 2022-12-17

**Authors:** Emilia Osmólska, Monika Stoma, Agnieszka Starek-Wójcicka

**Affiliations:** 1 Department of Power Engineering and Transportation, Faculty of Production Engineering, University of Life Sciences in Lublin, 20-612 Lublin, Poland; 2 Department of Biological Bases of Food and Feed Technologies, Faculty of Production Engineering, University of Life Sciences in Lublin, 20-612 Lublin, Poland

**Keywords:** intelligent packaging, sensors, biosensors, radio-frequency identification system (RFID), tags, nanotechnology, food packaging

## Abstract

The current development of science and the contemporary market, combined with high demands from consumers, force manufacturers and scientists to implement new solutions in various industries, including the packaging industry. The emergence of new solutions in the field of intelligent packaging has provided an opportunity to extend the quality of food products and ensures that food will not cause any harm to the consumer’s health. Due to physical, chemical, or biological factors, the state of food may be subject to degradation. The degradation may occur because the packaging, i.e., the protective element of food products, may be damaged during storage, transport, or other logistic and sales activities. This is especially important since most food products are highly perishable, and the maintenance of the quality of a food product is the most critical issue in the entire supply chain. Given the importance of the topic, the main purpose of this article was to provide a general overview of the application of biosensors, sensors, and tags in intelligent packaging used for food products. A short history and the genesis of intelligent packaging are presented, and the individual possibilities of application of sensors, biosensors, gas sensors, and RFID tags, as well as nanotechnology, in the area of the packaging of food products are characterized.

## 1. Introduction

The globalization and dynamics of the contemporary market, together with the excessive consumer expectations and preferences regarding safety (health and environmental) and product quality on the one hand and the stricter consumer law on the other hand, has led to the intensification of work on innovative concepts of packaging technologies and materials. In this regard, the safety of food products, understood as appropriate physical and sensory characteristics, microbiological safety, chemical composition, and nutritional value of the product, seems to be particularly important. It is known that many processes occur in raw materials, causing changes in their chemical composition and physical properties [1,2].

It should also be remembered that the consumer makes his choices based on perception and sensory expectations. Hence, in the case of inadequate freshness, smell, or appearance and other sensory characteristics, the consumer may reject such a product. It is known that many food products undergo various biological, chemical, or physical processes that ultimately lead to product spoilage [3]. Ecological aspects are equally important, as modern consumers are characterized by an increasing level of awareness, especially in the area of environmental protection and reducing food waste.

The deterioration of the condition of food resulting either from physical or chemical changes in the food itself or from the action of microorganisms may occur gradually during storage, transport, or other logistic and sales activities. Hence, it seems extremely important to constantly monitor certain qualitative features of the product during its transport and storage throughout the entire production and logistics chain. This is especially important since most food products are highly perishable, and the maintenance of the quality of a food product is the most critical issue in the entire supply chain [4]. It should be added that the aforementioned changes are difficult to estimate by consumers, especially in relation to packaged food. Moreover, often even a slight deviation from the norm, e.g., in relation to the color or consistency, results in discarding the product.

Therefore, packaging manufacturers have initiated activities aimed at creating so-called sustainable packaging, i.e., a product that is safe throughout its life cycle and is manufactured and recycled or biodegraded in a way that is beneficial for the natural environment. More specifically, packaging should support sustainable food management, i.e., solve the problem of food waste by preserving the quality of food products while ensuring their safety (preventing food-borne diseases and reducing the use of artificial additives) [5,6]. Modern packaging systems, including intelligent packaging, help to achieve this goal. They differ from traditional packaging in the ability to monitor and indicate the condition of products without having to comply with estimated expiry dates. It is a very practical solution in the case of food, medicines, or dangerous goods.

With regard to the food sector, the concept of intelligent packaging has also been developed, inter alia, to limit the destruction of food samples related to microbiological and chemical analyses and to modify the approach to a new dimension of food analysis [7]. On the other hand, the reasons for the growing importance of intelligent packaging in consumer purchasing decisions include deconsumption (i.e., limiting the amount of consumption in favor of its quality), lifestyle changes, constant striving to improve the quality of life, and the desire to protect the natural environment. In 2015, Tu et al. proved that the physical properties and improved aspect of food packaging containment are expectations that affect product sales and consumer attitudes [8]. Some even believe that the creation of “smart” packaging is a consequence of the introduction of intelligent technologies in most spheres of our lives: today most of the surrounding objects (cars, telephones, television, refrigerators, etc.) are or are to be smart in the near future. 

Intelligent packaging performs all the functions characteristic of conventional packaging on the one hand (above all, it protects the packed product); on the other hand, it can also acquire and process data. Hence, it protects goods even better and thus contributes to a positive consumer experience by, e.g., informing the consumer about the state of the environment of the packaged food. The idea of intelligent packaging is mainly manifested in the possibility of controling and measuring certain characteristics of the packaged food product [9].

It should be added that the terms “intelligent packaging” or “smart packaging” are very often used interchangeably. However, they do not mean the same thing. Obviously, these two terms refer to packaging systems used in the food, pharmaceutical, or cosmetics industries, as well as the packaging of other products that are quickly degraded, but it is important to note the differences between these types of packaging. In addition, active packaging can be distinguished among the packaging systems.

Intelligent packaging comprises all materials and products that are able to control/monitor changes taking place inside the packaged food. What is very important is that they provide information about the conditions of the packaged product but do not directly affect the packaged food. The main task of such packaging is to capture and provide data on changes in the quality of packed goods during transport and storage [10,11]. Another type of packaging systems are active packaging, which—through the content of various additives or substances—exert a positive effect on the extension of the quality and shelf life of a given food product. Nevertheless, these systems do not inform the consumer or the producer about changes taking place inside, unlike intelligent packaging. Therefore, active packaging plays a different role than providing an inert barrier to the external environment [12,13], whereas smart packaging is a combination of intelligent packaging and active packaging. Smart packaging has elements characteristic of intelligent packaging (it monitors changes) and active packaging (it influences these changes). What is more, smart packaging is able to provide comprehensive solutions in case of changes in the product [14,15]. This review presents information about one of the packaging systems, namely—intelligent packaging.

Intelligent packaging systems work in two main ways. The first is data carriers, i.e., various types of barcode labels or radio-frequency identification tags for storing and transmitting data. In turn, the second type of intelligent packaging is characterized by the presence of indicators or sensors in the packaging, which allow the monitoring of the external environment [7].

In contrast, Sohail [9] proposes a structure of an intelligent packaging system, as shown in Table 1, and concludes that there are three main types of tools used for intelligent packaging, namely:

1. sensors, mainly including:
  - gas sensors—devices monitoring changes in the composition of gases inside the package;
  - fluorescence oxygen sensors—detecting spoilage of the product, determining freshness of the product,
  - biosensors;
2. indicators—interactive indicators located outside or inside the package, which change their color and color intensity or expose dye dispersion under the influence of changes in the conditions prevailing in the package. In this way, they provide the consumer with qualitative or semi-quantitative information regarding the quality, freshness, and other parameters of the packaged product [16,17]:
  - time-temperature indicators—devices in the form of various types of stickers/labels attached to the outside of collective or individual packaging, recording even short-term thermal changes in the environment (an increase or a decrease in temperature beyond the limit acceptable for a given product) during the storage, transport, and distribution of food. TTIs can be based either on controlling physical changes or chemical reactions (e.g., melting, polymerization) or on changes in biological activity (microbes, spores, enzymes). These types of indicators are used primarily in the case of frozen products and chilled food, as well as in the packaging of semi-finished products intended for preparation in microwave ovens or ovens [16,18],
  - gas leakage/concentration indicators—devices used to control the tightness of packaging by detecting carbon dioxide without violating the integrity of the packaging material—a gas leak in the packaging may cause favorable conditions for the development of microorganisms,
  - microbial growth indicators reacting with microbial metabolites, which helps to observe the possible growth of microorganisms in the packed product,
  - freshness indicators—determining freshness by showing different colors or color changes. They provide direct information on product quality resulting from microbial growth or chemical changes in the food product. The microbiological quality can be determined by the reactions between the indicators contained inside the package and the metabolites of microbial growth, e.g., organic acids, ethanol, volatile nitrogen, biogenic amines, carbon dioxide, glucose, and sulfur compounds [19,20];
3. barcodes and radio-frequency identification (RFID) devices—devices that detect and identify a product in the supply chain by radio signal modulation.

Han et al. [21] indicate three main groups of tools used in intelligent packaging, based on the work of other authors [22,23]:

1. external indicators—attached to the outside of the package; they mainly include time-temperature indices and physical shock indices;
2. internal indicators—placed inside the package (in the upper part of the package or attached to the lid), e.g., oxygen leakage rates, carbon dioxide, microbial and pathogen indicators;
3. indicators facilitating a more effective process of information flow and communication of the packaged product with its consumer—special barcodes (storing information about food products, such as the use and expiry date, and enabling product traceability) and anti-theft, anti-counterfeiting, and anti-tampering devices.

Regardless of the technology used, however, the purpose of all such packaging systems is to provide the customer and the brand with the best experience and full control over the quality of the product. The sensors are able to track a wide range of indicators and thus provide customers and brands with valuable information on the basis of which it will be possible to make the right decisions: both consumer and strategic ones. It should also be mentioned that, in the future, this type of packaging may be one of the solutions in the field of the industrial internet of things (IIoT) by connecting to a network to share data on the condition of articles using sensors and software.

There are many publications available in the literature on various aspects and concepts of intelligent packaging systems, including the possibility of their application for various products. The research conducted by Vanderroost et al. [24], Ghaani et al. [20], Beshai et al. [25], and Chelliah et al. [26], who reviewed the solutions, features, and market potential of various types of intelligent appliances, including indicators, sensors, or RFID tags and barcodes, should be mentioned here. Other authors focused their deliberations on the application of the concept of intelligent packaging systems for specific types of food products: meat [16,18,19,27,28,29,30,31,32,33], fish and seafood [28,34,35,36,37,38,39,40], milk and dairy products [41,42,43,44,45,46], fruits and vegetables [47,48,49,50], juices [51,52], cheese [53], other fresh products [54], or chilled and frozen food products [55].

Hence, this article attempts to review modern solutions used in intelligent packaging in the context of their usefulness for various food products. The main focus was placed on tools based on sensors and biosensors, as well as RFID tags, as it is believed that these solutions may be most often used in intelligent packaging systems in the coming years.

Therefore, the purpose of this review is to determine the state of knowledge of the latest technological developments in the field of intelligent packaging systems in relation to food products, which are used primarily to maintain an appropriate level of quality and safety of protected goods. It also presents various concepts, classifications, and tools used in these systems for management of the supply chain of various foodstuffs.

The article is divided into the following sections: (2) genesis and essence of intelligent packaging, (3) intelligent packaging based on sensors, (4) intelligent packaging based on biosensors, (5) RFID—radio frequency identification systems, (6) hybrid intelligent packaging systems (7) application of nanotechnology in intelligent packaging systems. Finally, Section 8 presents the conclusions of the analyses in the form of challenges and perspectives regarding intelligent packaging systems.

## 2. The Genesis and Essence of Intelligent Packaging

The first proposals for intelligent packaging appeared almost 50 years ago, first on the Japanese market. Only 20 years later—in the 1990s—other markets, mainly European and American, became interested in these systems. More intensive development in this area in the European Union countries could be observed only after the entry into force of the framework regulation of the European Parliament and of Council No. 1935 of 2004 [56]. It should be added that, in the first years of functioning, intelligent packaging was mainly used in the pharmaceutical industry and then also found application in the food industry [57,58]. It is also important that there was no legal framework for intelligent packaging in the EU for many years. Moreover, due to the stricter EU law, US intelligent packaging systems cannot be easily introduced to Europe [59].

Initially, intelligent packaging was defined as a type of packaging that could sense and inform users about environmental changes [60]. A few years later, Han, Ho, and Rodrigues [21] divided intelligent packaging into two categories: simple intelligent packaging and interactive or responsive packaging. There were also other definitions that pointed out in particular that such packaging systems contain various types of devices and sensors that are capable of detecting and providing information about the functions and properties of the packaged food, e.g., informing consumers that a product is damaged [21,61] and/or include an external or internal indicator for active product history and quality determination [62].

On 29 May 2009, the Commission Regulation (EC) No. 450/2009 was published to regulate the issues related to the safety of the use of active packaging materials [63]. It defines intelligent packaging as “materials and products that monitor the condition of packaged food or its environment”, which means that their main purpose and task is to provide the consumer with reliable information about the conditions in which a given food is stored. The regulation also defines the specific requirements for the market circulation of active and intelligent materials, as well as materials intended to come into contact with food [64]. Packaging is in direct contact with the food it protects. Therefore, all materials and products must be controlled in terms of their safety, as substances contained therein can migrate inside the food [65].

The European Food Safety Authority (EFSA) supervises food safety by accepting and publishing scientific opinions. The threat that may occur in the area of quality and safety of substances used or intended for the production of materials that come into direct contact with food is carefully assessed. EFSA also analyzes safety in the context of related packaging production processes, e.g., the recycling of plastics. In addition, EFSA publishes several papers or protocols each year that allow the assessment of consumer health risks [66,67]. 

Regarding the aforementioned Regulation EC 450/2009, it should be added that it contains an updated list of substances that are safe for the production of such packaging. The list is available for all European Union countries. EFSA’s Panel on Food Contact Materials, Enzymes, Flavorings, and Processing Aids (CEF) acts as a supervisor in this context, i.e., it analyzes all substances on the list. These substances are added to the list only after their safety has been assessed [68,69].

Contemporary literature offers many different definitions of intelligent packaging. As reported by Bagchi and He [7], intelligent packaging is defined as a packaging system that is capable of performing intelligent functions to facilitate decisions leading to an extension of shelf-life, increasing safety, improving quality, providing information, and warning about possible problems. Among these functions, the authors mention detecting, sensing, recording, tracing, communicating, and applying scientific logic. 

In turn, as suggested by Yam et al. [70], the role of intelligent packaging is to facilitate communication throughout the supply chain, to facilitate the recording of changes in the internal and external environment, and to inform users about the condition of packaged food products, so that appropriate actions can be taken to achieve the desired benefits in terms of improving food quality and safety. They add that this is done by small labels or tags that are attached to the primary packaging (e.g., pouches, trays, and bottles) or more commonly to the secondary packaging (e.g., shipping containers).

Yet another definition is given by Brockgreitens, i.e., “IP is any type of container that provides a specific functionality beyond the function of a physical barrier between the food product and the surrounding environment”. In turn, Biji states that IP are packaging technologies, which, using internal and external indicators, monitor the interaction between food, packaging, and the environment. He also adds that the knowledge of product quality, packaging, or the environment is extremely important, as it forms the basis of the bond of responsibility throughout the entire food supply chain (storage, transport, distribution, and sales).

Intelligent packaging has the ability to monitor specific parameters (internal and/or external product environment); these systems are therefore able to provide consumers with certain information about the packaged product, without having to open the package itself. This information is mainly related to quality and safety and may indicate changes and irregularities, e.g., in temperature, oxygen, or carbon dioxide content occurring during the storage and distribution of food [9]. Importantly, even the most processed food is still a biologically active system: it oxidizes, gives off gas, and changes color or humidity [10]. This is possible by placing appropriate interactive indicators outside or inside the package. They occur mainly in the form of color indicators that change their color under the influence of changes in the conditions prevailing in the packaging. However, it should be remembered that they should not come into direct contact or interact with food [11,71].

The statement that intelligent packaging is a system that can monitor the quality/safety status of a food product and provide an early warning to the consumer or food producer seems to be common to all definitions. Its main goal is therefore to improve the efficiency of transport and to ensure control and secure transported goods. On the other hand, a secondary goal may be to support marketing or logistic activities. Therefore, increasing numbers of companies are introducing new solutions in the field of intelligent packaging to the market.

It seems that intelligent packaging is very useful for both industry and consumers, especially in terms of quality assurance and food safety. The sensors or devices on the packaging that are able to share relevant information from the packaging system provide up-to-date information on the condition of the food. Intelligent packaging can also often provide its users with certain information about the process of the production, distribution, and storage of the food product and about its ingredients and properties [7,9]. In addition, it gives the opportunity to control the quality characteristics of packaged food. It should be added that the constant monitoring not only reduces food waste but also protects consumers against potential food poisoning; in addition, attention should be paid by businesses to maximization of the efficiency of the food industry and the improvement of traceability [10,72]. 

Hence, it seems that intelligent packaging systems have great potential and should be used on a large scale to improve product safety and reduce the negative impact on the environment on the one hand and increase the attractiveness of the packaged product and food industry companies on the other hand.

## 3. Intelligent Packaging Based on Sensors

A sensor is an instrument that detects, locates, or determines events or changes in the surrounding environment and then transmits signals to measure physical or chemical parameters. Generally, sensors are composed of a receptor (whose function is to transform physical or chemical information into a form of energy) and a transmitter (which converts the energy into an analytical chemical, optical, electrical, or thermal signal) [54,73]. On the other hand, intelligent sensors or smart sensors are equipped with dedicated signal processing functions in order to increase the flexibility of designing sensor devices and to implement new detection functions [74].

With the current development of science, there are many structures on which the functioning of sensors is based with great potential for use in intelligent packaging. 

Devices based on a chemical sensor operate on the principle of transforming the chemical information flowing from the environment into an operative measurement signal. A characteristic feature of chemical sensors is their ability to selectively capture a substance or an ion. The analytical signal is formed from the adsorption of the actual analyte in the recognition layer. Energy conversion is combined with analyte detection, resulting in a change in receptor properties in terms of redox potential, pH, temperature, or light [20]. Moreover, chemical sensors react to the selected analyte through a reversible chemical interaction, which facilitates its qualitative and quantitative determination [20]. Thus, they are the best alternative to time-consuming analytical instruments like gas chromatography–mass spectrometry (GC–MS), which can be used only by compromising the integrity of the food package [20].

There are two common chemical elements of the sensor action. One of them, i.e., the receptor (the sensing part) selectively capturing a given factor in the area where a chemical reaction takes place, and the other is the converter, i.e., the transduction part. The converter helps in converting one form of energy into another and transferring the detail into an analytical signal. The emerging chemical reaction generates a signal manifested as a color change, fluorescence, heat release, or a change in the frequency of the crystal oscillator [20,75].

As suggested by Hanrahan et al. [76], an ideal sensor should have five main attributes: (1) selectivity or specificity to the target species; (2) vulnerability to changes in target species concentrations; (3) quick reaction time; (4) prolonged shelf life, at least a couple of months; and (5) compact dimensions, with the capability for cheap production. 

**Figure 1:**
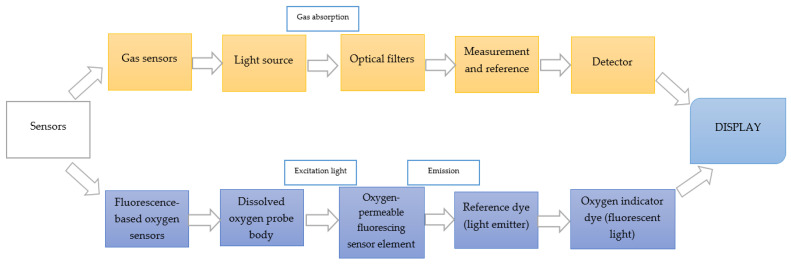
Various types of sensors used in intelligent packaging [54].

There are various types of sensors that test different parameters. One of their divisions is presented in Figure 1.

In addition, to ensure the highest possible level of food safety, sensors that measure oxygen and carbon dioxide (gas sensors), pH change, humidity, time, temperature, specific chemicals such as TVBN, chemical array, or bacteria deserve special attention. These indicators represent the physical and nutritional status of the food, which is a key element illustrating its quality and edibility [77]. 

Several factors that are measured in intelligent packaging are characterized below in order to determine whether the product has retained its quality features and safety level.

### 3.1. Sensors Based on Oxygen and Carbon Dioxide 

Among the most common solutions in the field of intelligent packaging systems based on sensors, one should undoubtedly mention gas sensors, as the deterioration of food products is identified on the basis of emitted gases, such as CO_2_ or H_2_S. 

Gas sensors are used to monitor changes in a package caused by various external stimuli (e.g., environmental conditions). Very often, such devices are able to determine the effectiveness of the active ingredients in the packaging, the so-called O_2_ and CO_2_ scavengers [78]. Gas sensors react quantitatively. Due to their transient state, the presence of gas can be detected by changes in the physical parameters of the sensor [10].

Carbon dioxide is known for its antimicrobial action against certain selected microorganisms; therefore, it is very often used in packages with modified atmosphere. The use of such a solution helps to extend the shelf-life of the product [79]. It is also worth noting that some materials are more permeable to CO_2_ than oxygen; consequently, this gas is dissolved in food. Thus, a very important device to stop these unwanted effects is the carbon dioxide generator, which helps to stabilize the concentration of this compound and prevents the deformation of the package [80].

CO_2_ sensors are mainly based on non-dispersive infrared. However, they can be sensors operating on the principle of chemical transformations as well. Very importantly, NDIR sensors collect measurements of CO_2_ content by means of the absorption of the gas at a certain wavelength, which makes them spectroscopic sensors. Chemical sensors that monitor CO_2_ levels function by working with polymeric or solid electrolytes. In contrast, infrared sensors are used to detect O_2_, as well as systems based on ultrasonic, laser, or electrochemical transformations [81,82].

The quickest and easiest way to observe changes in packaging is to use redox-based colorimetric indicators. The compounds used in such indicators are sodium or potassium hydroxide. To detect the presence of oxygen, which is responsible for the microbial spoilage of food, a colorimetric indicator using methylene blue and a reducing agent is used (e.g., glucose) [83,84]. 

Gas sensors include amperometric oxygen sensors, potentiometric CO_2_ sensors, semiconductor metal oxide transistors, piezoelectric crystal sensors, and organic conductive polymers [10,19].

However, it should be remembered that the reducing agent is susceptible to the action of oxygen, and therefore these indicators must be kept in anaerobic conditions to prevent undesirable reactions that may consequently cause the indicator to malfunction. The questionable safety of a food product packed in a package with such a sensor may also be a big problem. Particular attention should be paid to the accidental contamination of food with a harmful synthetic component of the sensor [42].

### 3.2. Sensors Based on pH Changes and Specific Chemicals 

Changes in pH and the formation of specific chemical compounds, often harmful to humans, are associated with the development of pathogenic microorganisms that cause food spoilage. A similar scheme of operation as in the case of O_2_ and CO_2_, i.e., colorimetric tests, are used in the process of controlling changes in pH and the formation of specific chemical compounds. Such tests are most often built into the packaging, so that the user can observe the changes in a simple and quick way.

An interesting solution seems to be the polyaniline foil indicators designed for perishable fish by Kuswadi et al. [85]. The scientists have created a packaging that changes color in response to the appearance of amine compounds as a result of deteriorating food quality. 

To detect changes in pH, electrochemical sensors are very often used, the operation of which is based on the redox reaction on the electrode surface. It releases an electrical signal that is able to detect volatile amine compounds formed during food oxidation. This is especially helpful for animal products, including meat and fish [86,87].

Bhadra et al. [40] constructed a passive pH sensor covered with a special hydrogel facilitating the determination of the concentration of volatile compounds. The entire device uses the effect of a resonance frequency, which changes depending on the concentration of volatile amines appearing in a given environment. Any changes in the above-mentioned parameter can be detected by controlling the impedance of the outer coil coupled to the sensor. 

One of the main advantages of such sensors is the speed and sensitivity of decay process detection; moreover, these sensors provide very important information regarding the concentration of the measured harmful compound. Additionally, they are more advanced than classic colorimetric tests. Some disadvantages appear already when such a system is implemented in packaging systems, as it is much more complicated than color measurement [20].

### 3.3. Sensors Based on Humidity

Food moisture can often be a major problem for ensuring food quality and safety throughout the supply chain. Products such as meat, fish, fruit, and vegetables are characterized by high water activity and therefore often lose moisture and become dry [88]. On the other hand, products with low water activity usually absorb moisture from the environment, which results in the deterioration of their quality, including organoleptic characteristics [87].

Hence, it is advisable to control the humidity level inside the package in real time. Various approaches are currently being used to monitor this parameter; these include measurements of changes in the dielectric properties, capacitance, and resonant frequency of crystals. Protein, which is sensitive to humidity, is very often used for intelligent packaging that measures humidity. 

An example of such packaging is nanocomposite foil, which has a layer based on gelatin due to its dielectric properties; additionally, this foil has a ZnO layer, which is intended to detect the signal [86,89].

Bibi et al. [90] used wheat gluten to detect changes in relative humidity in packages. The principle of operation is very similar to that of the nanocomposite foil, i.e., gluten was used due to its dielectric properties.

Intelligent packaging also very often uses sensors that are placed inside the materials used for their production. The interlayer electrode (IDE) acts as an induction layer in packages made of biodegradable materials. The ambient relative humidity function defines the capacitance between electrodes. It should be added that the dielectric constant intensifies with the absorption of moisture from the atmosphere [91]. Besides, the increase in humidity inside the package makes the specially designed layer (usually inductive) absorb the vapor with water. A result of this process is the modified capacitance of the capacitor and resonant frequency. A composite of starch and polypropylene (PP), glossy photo paper, polyvinyl alcohol, polylactic acid (PLA), and planarized polyethylene terephthalate (PET) are used to manufacture this type of sensor [92].

Moisture-prone products are a special case wherein intelligent packaging has a wide range of applications. They are especially useful for dried products such as flakes, powders, and cereals, as well as salty and sweet snacks, as the water activity in this type of product is much higher, and thus their quality characteristics may deteriorate.

### 3.4. Sensors Based on Time and Temperature (TTI)

Temperature and time are two very important factors for food freshness, safety, and quality. Most food products have an assigned use-by date and storage conditions (refrigerated, room temperature). It is also very difficult to determine whether a given product has been stored properly [93]. Temperature is a key element affecting practically all quality parameters; therefore, it is very important to monitor it and to react in crisis situations. Time-temperature sensors allow the real-time batch control of consumer products throughout the supply chain [94].

For example, Evigence Sensors Inc, based in the United States, has time-temperature sensors for sale. The company ensures that their TTIs are relatively cheap and automatically activated, so they can be used on production lines, they have a visual and digital indicator, which makes it easier to read (e.g., using a smartphone), and most importantly, they are not susceptible to degradation due to exposure to the sun or UV radiation. 

The methodology of the TTI sensor operation is based on some kind of reactions taking place between two or more substrates, which results in a peremptory color of the indicator as a result of the dominant reaction [95].

Nowadays, TTI can be divided into several varieties according to the nature of the reaction. These include:

chemical TTIs working by polymerizing 1,4-additive monomers under the influence of high temperature. In this way, polydiacetylene compounds (PDAs) are formed. It is very important that this reaction is irreversible, and the rate of reaction will be proportional to the subsequent increase in temperature. Additionally, they are also based on photoluminescence by the principle of thermally induced decay in the reverse reaction of photoluminescent compounds. The reaction process will depend on the increase in time and temperature, as a result of which the degree of the fading of the indicator will be visible on the package [94];

physical TTIs; their operating principle is based on diffusion, nanoparticles, and electrons. Corresponding changes are created depending on the reaction temperature or the solid-to-liquid transition temperature. Thanks to these dependencies, the color of the indicator changes. While absorbing heat, a sensor based on the use of nanoparticles with thermochromic properties changes the surface of metal nanoparticles (Ag NPs/Au NPs), as a result of which the wavenumber migrates to the visible surface, thereby changing the color [96];

enzymatic TTIs use the hydrolysis reaction between the enzyme and the substrate in the indicator, which yields the appropriate color. Depending on time and temperature, the enzymatic reaction becomes more intense, revealing a color consistent with the intensity of the exposure to these two factors. Given such indicators, it is possible to control various types of food depending on the selected enzymes, substrates, activators, or inhibitors [97];

microbial TTIs base their action on metabolites produced by microorganisms in appropriate environmental conditions (according to time and temperature) to change the indicator element. Nowadays, yeast or lactic acid bacteria are most often used due to the anaerobic acid production. This results in a color change in the indicator [94].

There is a growing body of information about TTI indicators composed of self-generating materials. For example, Choi et al. [98] created an elastic fiber from aromatic disulfide based on thermoplastic polyurethane (TPU). This nanofiber is opaque and becomes transparent with time and changes in temperature. Additionally, the built-in display facilitates temperature control. In addition, this material has high resistance to mechanical effects.

### 3.5. Characteristics of Optical Sensors and Others 

Optical sensors are becoming an increasingly frequent subject of research due to their great potential to be use in food packaging. Such sensors have been proved to be less expensive than others. Very importantly, they do not need electronic connections and additional devices to function properly. 

Besides, they can be used by untrained operators. The principle of their operation is mainly based on the following systems: photonic structures and plasmonic particles, fluorogenic receptors, or simply a colorimetric test [99].

Examples of sensors that are used in intelligent packaging systems include those already described by Baleizao et al. [100]. The research team developed a highly sensitive dual optical sensor to detect oxygen at different temperature levels. It consists of two light-emitting compounds, one of which can be used to detect temperature and the other to detect oxygen. The results obtained by the authors confirmed that the double sensor was able to detect temperature changes in the range from 0 to 120 °C.

In the following years, new ideas for this type of sensors that could be used in the packaging of food products were developed. For example, Borchert, Kerry, and Papkovsky developed an optochemical sensor to measure CO_2_ in food stored in modified-atmosphere packaging. They found that the sensor could retain its sensitivity to CO_2_ at 4 °C for almost three weeks [50]. Heising, Boekel, and Dekker have designed a sensor to check the freshness of packaged cod fillets by monitoring volatile compounds released from the fish during storage [41]. 

In addition, there are also luminescence sensors in which emitted fluorescence or chemiluminescence signals are measured after the analyte is retained in an appropriate carrier. Then, the reaction of solid phase luminescence (SPL) or its equivalent (SML) begins. This reaction helps to detect the desired food ingredients and verify the existence of contaminants in a given product [101].

The most promising indicators are sensors that are sensitive to pH changes caused by food product degradation, such as acid or alkaline gases [102]. Consideration should also be given to fiber optic oxygen sensors based on fluorescence. Unlike conventional gas sensors, they do not utilize oxygen, are not susceptible to electromagnetic interference, and are additionally suitable for measuring liquids and gases. The most popular fluorescence-based oxygen sensor on the market is O_2_xyDot from OxySense in Delware, the USA. Its main advantage is that it can be read from the outside of the package without destroying it [16]. However, it should be mentioned that this system can only be used in transparent packaging, which is a limitation of its application.

The main advantages of the characterized sensors include their high measurement precision and sensitivity. In addition, they provide quantitative information on temperature, humidity, and gas concentration inside the package. In addition, the sensors can be linked to various IP systems such as RFID. Consequently, the user receives more accurate information [2]. 

## 4. Intelligent Packaging Based on Biosensors

Intelligent packaging with biosensors, which is a special case of sensors, also deserves special attention. There is a significant difference between chemical sensors and biosensors in the recognition layer. Chemical sensors consist of a layer designed to recognize the chemical compound. In contrast, biosensors are composed of receptors of biological materials, i.e., antigens, enzymes, nucleic acids, or hormones [103].

Biosensors work on the principle of monitoring and detecting potential changes (biological reactions) in controlled products. In addition, such a biosensor becomes an integral part of the packaging or is located directly within it. For example, there is packaging that signals the onset of putrefaction processes within the package or baby food packaging that contains sterility sensors (based on amylase detection).

**Figure 2:**
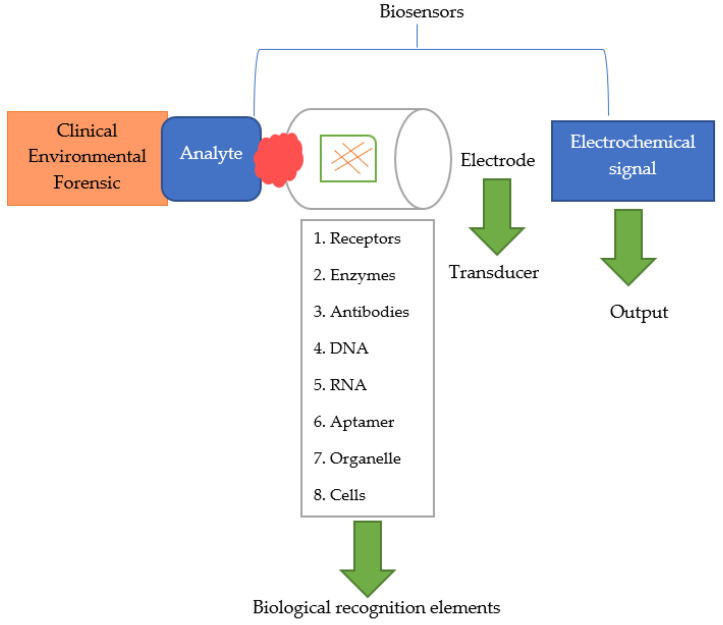
Theory of operation of biosensors [26].

**Figure 3:**
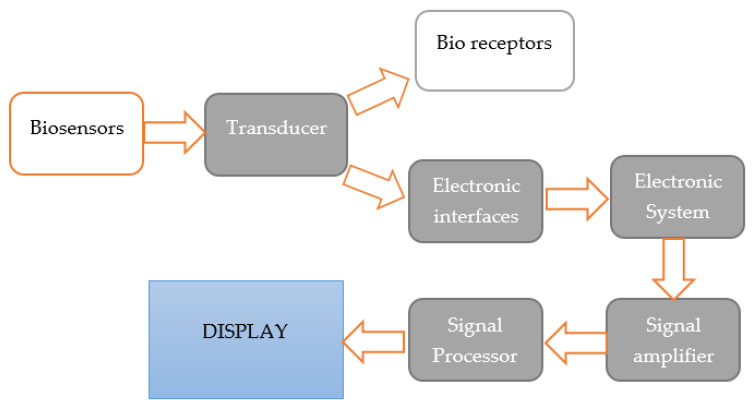
Mechanism of operation of biosensors [54].

The main component of biosensors is the bioreceptor, which is responsible for controlling microbiological changes in food products. In addition, it is capable of recognizing enzyme activity, antigens, hormones, or microorganisms. In addition to the bioreceptor, there is also a transducer (acoustic, optical, or electrochemical), which converts biological signals into electrical ones [19,20]. The operation of a typical biosensor is shown in Figure 2 and Figure 3.

Today, biosensors are increasingly being used by manufacturers. In the food packaging industry, and especially in the fish and meat industries, biosensors are being used in pathogen detection and safety systems [104]. 

SIRA Technologies (USA) has developed the Food Sentinel System biosensor. The technology is based on barcodes found on packaging; a specific antimicrobial antibody is introduced into the membrane-forming part of the barcode. When the product has been exposed to unfavorable conditions and a pathogen enters the product, the biosensor attaches to the membrane-forming barcode alerts consumers and retailers that the product is unfit for consumption. When the product becomes contaminated, the biosensor turns red and a dark bar is created, making the barcode unreadable when scanned; consequently, this prevents the sale of the defective product [20].

Another solution for monitoring the safety of food products by biosensors has been proposed by Toxin Alert Inc. The Toxin Guard™ technology is based on an imaging diagnostic tool used to detect pathogens or other microorganisms that pose a risk of contaminating food products. The device is excellent at detecting such microorganisms as *Campylobacter* spp., *Escherichia coli* O157:H7, *Listeria* spp., or *Salmonella*. Toxin Guard™ works by reacting antibodies with antigens on polymeric packaging films—the antimicrobial antibodies are directly integrated into the plastic food packaging. This device works on the principle of an immunoassay. Moreover, when pathogenic bacteria are present, the bacterial toxin fuses with the antibodies contained in the device, and a clear change in the color can be seen on the flexible polymer (polyethylene) film; this means that a positive result is indicated by a visual signal [105].

Ma, Du, and Wang developed a biosensor by integrating curcumin (Cur) into a tara gum (TG)/polyvinyl alcohol (PVA) film. The color response was visible within 1–3 min in the NH3 environment. The team conducted their research on shrimp; they were able to obtain positive results, indicating that the film could be used as a sensor in the food industry [106].

Another biosensor is capable of detecting xanthine, which is a degradation product of adenine nucleotides in animal tissue. For this purpose, xanthine oxide is inactivated on platinum, silver, or pencil-graphite electrodes [71].

Another example, i.e., a device designed by Flex Alert (Canada), is a widely available flexible biosensor for discovering pathogenic compounds in finished food. Very interestingly, this system is capable of inspecting food throughout the supply chain. Flex Alert’s flexible biosensors detect *E. coli* O157, *Listeria* spp., and *Salmonella* spp. pathogens or aflatoxins [24].

## 5. RFID—Radio-Frequency Identification Systems

Temperature deviations during transport and storage continue to cause significant food losses. A large part of these losses could be avoided if information on the differing transport conditions and the resulting changes in the shelf life of packaged food were available in real time. In particular fresh food is quickly degraded; therefore, in order to control its quality and safety at every stage, the use of radio frequency identification systems seems to be the right solution, as it allows controlling food products almost throughout the supply chain [107].

**Figure 4:**
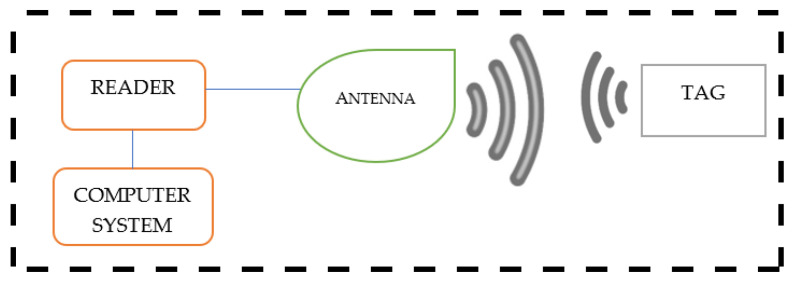
Mechanism of operation of RFID—diagram [111].

A radio-frequency identification (RFID) system is a technology based on the integration of tags (made of microchips connected to a small antenna), readers (capable of discharging radio signals and accepting answers from the tag in reply to the sent signals), middleware, and an application system, which is used to integrate the entire technological structure (Figure 4). This system can be placed on any material (e.g., film, paper) and can have any shape. RFID tags are the most advanced data carrier systems and are attached to the package to track and identify the object using radio frequency electromagnetic fields. Tags and readers are designed to communicate with each other. The most advanced RFID systems have the ability to accept data from a distance of 100 m, with a storage range of 1 MB. In addition, a dedicated IT system includes a function to control all readers using middleware and is able to read data records directly from tags [108,109,110]. 

It should be added that tags have the ability to collect, store, and transmit information in real time to the user’s information system without contact and out of sight. This is possible because they can be linked to an article, box, container, or pallet. Thus, they represent an advanced form of support data information facilitating product identification and location.

Depending on the power supply for communication and other functions, RFID tags can be divided into four basic types: active, passive, semi-active, and semi-passive. Active RFID tags have a reading range of 91 m or more and have a battery that allows them to communicate autonomously. Passive RFID tags, on the other hand, have no internal power supply (no battery installed); therefore, they are unable to communicate until the RFID reader’s emission is activated. RFID systems can also be classified according to the frequency range used: low frequency (LF), 125 to 134.2 KHz; high frequency (HF), 13.56 MHz; ultra-high frequency (UHF), 868–956 MHz; ac frequency, or 2.45 GHz microwave frequency [2].

The task of RFID technology is very simple—it involves automating the transmission of information about a product to a database. Using radio waves, this system helps to identify objects and to wirelessly encode and transmit information data [112]. This allows constant access to the messages contained in the carrier.

On the basis of RFID, a traceability information system is created that combines the logistics process of the supply chain, elements of the company’s business structure, and food safety management. With this solution, the manufacturer is able to monitor information on all types of food, as well as update and store this data in real time. Ultimately, it is also possible to streamline the logistics structure; to guarantee food quality and safety to consumers; and finally to make it easier for businesses, consumers, and regulatory departments to track and provide logistical guarantees for food quality and safety [113].

The creation of a global system containing general information on food products undoubtedly facilitates the flow of data from the manufacturer to the consumer. As consumers are becoming more aware, manufacturers are introducing systems relating to traceability and food traceability [114].

For many years, the RFID system has been implemented in various industries due to the multitude of emerging benefits. These can include, among others, control of food safety, guarantee of reliable and high quality food products, reduction of expenses for quality verification, minimization of costs occurring in the logistics process, fast and efficient monitoring of transmitted information, and reduction of insurance and liability costs [115].

Very often, temperature sensors are added to RFID tag in a package in which a food is contained; hence, it is additionally possible to control the temperature at the right time in the supply chain execution procedure [116]. Interestingly, modern RFID systems are able to read all of the individual measured temperature data from the entire period covered by the measurement. Thanks to this function, the device counts statistical changes and communicates them to a computer so as to verify their compliance. Such a solution greatly facilitates the control of the entire logistics system of the logistics chain.

RFID technology has found its way into food packaging due to its ability to include a lot of information about products. An example of the application of this technology is the SmartCorq wine stoppers from Arnoldo Caprai, which included data on the type of wine or its country of origin and additionally include information on its processing and storage conditions [117].

Another example was presented by Papetti et al. [53], who developed an electronic control system combined with a quality monitoring system for traditional Italian cheese. Interestingly, consumers have access to all of the important information, which is very easy to obtain using an RFID code. In turn, Rahman designed a wireless network of RFID-controlled sensors to control wine ingredients throughout the supply chain. The performance and efficiency of such a system proved to be very high; hence, the efficiency of the real-time tracking proved to be high [118]. In contrast, Potyrailo et al. [119] proposed a systematic real-time assessment of packaged milk freshness, marketing, and distribution using RFID tags.

Avery Dennison has developed an RFID tag called WaveSafe that can be used in microwaves [19]. A few years ago, Amin et al. also developed a unique chipless RFID sensor system for the wireless detection of food and other tagged items. Due to the lack of chips, the RFID sensor does not require an electrical power source like other RFID systems, allowing it to be easily applied and used without any maintenance requirements [120].

## 6. Hybrid Intelligent Packaging Systems

Nowadays, it is becoming increasingly common for manufacturers of modern intelligent packaging to use a combination of the basic tools in these systems based on scientific research. Mention should be made here of the work carried out by Huang et al. [121], who developed a pH sensor embedded in a battery-free radio frequency transmitter for monitoring degradation processes in fish products. Also in the same year, Smits et al. [122] have proposed an RFID tag with sensors capable of measuring temperature, humidity, and the presence of volatile amine compounds to assess the freshness of cod.

Sen et al. have developed a system containing an RFID tag combined with a temperature sensor, a gas sensor, a reader, and a server to monitor the freshness of meat (pork) [123]. This monitoring system successfully demonstrated the freshness of meat for four classes, i.e., high, medium, low, and spoiled. In contrast, Eom et al. used an RFID + CO_2_ and O_2_ sensor combination to determine the freshness of broccoli [49].

Martínez-Olmos et al. based their solution on an RFID tag, which they integrated with an optical oxygen indicator containing a layer of platinum octaethylporphyrin. They showed that the system they created is suitable for use in food packaging systems with an oxygen concentration lower than 2%, a detection limit of 40 ppm, and a resolution of 0.1 ppm O_2_, with electricity consumption of 3.55 mA [124].

Lorite et al. [4] have developed a novel and functional intelligent critical temperature indicator (CTI) integrated with RFID. The indicator is based on the melting point of a specific solvent and was developed based on microfluidic technology. As a result, the CTI sensor combines irreversible visual color changes and radio-frequency identification (RFID) technology to facilitate real-time supply chain monitoring through the simple use of an RFID reader at strategic points.

In turn, Alfian et al. [125] proposed an integrated food traceability system using RFID technology and a wireless sensor network to collect temperature and humidity during storage and transportation throughout the kimchi supply chain in Korea. Validation of this system yielded positive results for detecting product location in real time and providing a complete temperature and humidity history [125].

## 7. Application of Nanotechnology in Intelligent Packaging Systems

The concept of modern nanotechnology was first established in 1959 by Nobel laureate Richard P. Feynman during his lecture “There’s Plenty of Room at the Bottom” [126], in which he presented the novel concept of manipulating matter at the atomic level. Then, in 1974, Japanese researcher Norio Taniguchi was the first to use “nanotechnology” to describe semiconductor (precision engineering) processes occurring at the nanometer level (i.e., with tolerances of a micron or less). In his definition, he claimed that nanotechnology involves the processing, separation, consolidation, and deformation of materials by a single atom or molecule [127,128]. Another definition says that nanotechnology means understanding, controlling, and restructuring matter on the order of nanometers (i.e., less than 100 nm) to create materials with entirely new properties and functions [129]. It should be added that the concept of nanotechnology varies from field to field and from country to country. On October 18, 2011, the European Commission (EC) adopted the “Recommendation on the definition of a nanomaterial” [130].

Since Feynman’s lecture, there have been many revolutionary discoveries in various fields of science concerning the manipulation of matter at an extremely small scale at the level of molecules and atoms, i.e., at the nanoscale. Hence, the term nanotechnology is commonly used to describe anything that is very small—nanostructure materials have a mean size of 1–100 nm [131,132]. It should be added that, regardless of the definition used, nanotechnology uses nanomaterials for the benefit of humans [131].

Currently, nanotechnology is widely used in many different sectors—manufacturing, electronics, medicine and healthcare, energy, biotechnology, information technology, national security, agriculture, and the environment [133,134]. This is related to the fact that innovative nanostructured materials and nanocomposites are known for their unique physical and chemical properties and enhanced performance and are therefore preferred over their macrostructural counterparts. Hence, they are also used in the food industry not only for food preservation and processing but also for food packaging [132].

It is undeniable that, in the context of food, packaging is one of the most essential and difficult elements, as food products must be safe and must have the proper quality for a long time. Hence, manufacturers should pay very close attention to choosing the right packaging [135]. Intelligent packaging incorporates nanosensors to monitor the condition of food or other contents. Nano-enabled products include nanocomposites, barrier layers, active components, and intelligent product features [136]. Nanomaterials and nanoparticles include nanoparticles, nanotubes, fullerenes, nanofibers, nanocylinders, and nanosheets [137].

Nanocomposites used in packaging films are an example of an interesting solution. They overcome the problems of standard packaging because they are enriched with antimicrobial properties. They prevent degradation from the outside and protect the product thermally due to the presence of nanosensors that alert consumers to the conditions and safety of the food product [138].

The structure of packaging containing nanoclay platelets slows down the transmission of gases and thus significantly extends the shelf life of stored food and beverages. Nanocomposites containing nanoclays improve the strength and flexibility of packaging materials while providing effective barrier properties [136,139,140]. Other nanocomposites include chitosan-based nanocomposites [141,142,143,144], proteins [139,145,146], cellulose [147,148], and starch [149,150,151]. In addition, various metals and their oxides, e.g., silver nanoparticles (Ag NPs), nano zinc oxide (nano-ZnO), nano titanium dioxide (nano-TiO_2_), etc., have been used for antimicrobial activity [138].

It should also be mentioned that, thanks to their unique optical and electronic properties, nanomaterials contribute to the development of a new generation of electronic devices, for example, nanotransistors for the construction of future nanoprocessors and nanomemories, nanobatteries, and nanosensors [2]. Of particular note in the context of intelligent packaging systems are nanosensors. The development of these ultra-sensitive biosensors is made possible by nanomaterials due to differences in their external-surface-area-to-volume ratio, surface characteristics, ionic conductivity, and excellent stability [26].

The application of nanosensors for food packaging, compared to conventional packaging, generates such benefits as an increased speed of analysis, a higher level of sensitivity, and an increased sample throughput (multiplexed systems) on the one hand and lower complexity of tests and reduced financial outlays on the other hand [133]. Nanosensors embedded in packaging are used to detect pathogens and contaminants; in addition, microbial coatings protect contents from bacteria [152,153,154]. Sensors can be referred to as nanosensors when their size, sensitivity, or the distance between the analyte and the sensor is within the nanoscale [138].

The following nanosensors have been devised and used in intelligent packaging: nanosensors in raw bacon packaging for oxygen detection [155], an electronic tongue consisting of an array of nanosensors extremely sensitive to gases released by spoiled food [156,157], fluorescent nanoparticles detecting pathogens and toxins in food and crops [158,159,160,161,162,163], nanosensors detecting temperature changes [164,165], nanosensors detecting organophosphorus pesticide residues in food [166,167,168,169,170,171,172], and nanosensors detecting changes in humidity or temperature under the influence of moisture [173,174].

Nanotechnology can also provide additional functionality to barcodes. This is done by incorporating nanoparticles into the barcode ink, as they can provide tracking and traceability information. The RFID tags then consist of a silicon microprocessor providing memory for data and a coupling element acting as an antenna to access the information [136,175,176,177].

In conclusion, the implementation of nanotechnology in various areas of food packaging is undoubtedly a growing field. Through the use of nanosensors in packaging, the new technology helps to increase food safety, reduce the time of detection of pathogens or control the quality of food and packaging throughout the supply chain and at various stages of the logistics process, and provide a longer shelf life for food products. Nanotechnology applied to intelligent packaging can help provide the authentication, tracking, and localization of product features to avoid the adulteration of a variety of products intended for a specific market. It should be added that it can contribute not only to providing end users with improved products but also to better consumer experiences and brand recognition.

## 8. Challenges and Perspectives

Intelligent packaging innovations are an area of interest for researchers and manufacturers due to the range of benefits they offer. The use of intelligent packaging guarantees higher food quality and safety, in addition to convenience for consumers, but also provides control throughout the production and logistics chain.

The future of intelligent packaging applied to food seems optometric, but there are some gaps, and more perfect research needs to be done to make intelligent packaging profitable. This would make them an everyday part of food packaging. What is needed is a presentation of the economic side, as well as the technical side, which are integrally related to the feasibility of various new systems. This is very important from the perspective of manufacturers introducing such technologies on a large scale. This is achievable with the cooperation of governmental bodies, research centers, and companies.

The comprehensive description of the applied technologies in this article can help researchers, manufacturers, marketers, and consumers to tailor an integrated packaging system to individual applications.

Noteworthy, for the time being, intelligent packaging is mainly used in the food industry and less frequently in other industries. It is worth considering other areas of life sciences wherein intelligent packaging could prove its worth in e.g., the pharmaceutical or cosmetics industries. The safety level of medicines or cosmetics should be as high as the safety of products intended for consumption. As in the food industry, barcodes would improve the traceability or temperature control of such products.

As mentioned earlier, issues related to the price of intelligent packaged products need to be addressed. The higher price of such products may effectively deter potential consumers. Understandably, consumers do not want to pay more for their favorite products only because they come in different packaging. Hence, the advantages of such systems need to be communicated. Very importantly, trust needs to be built among consumers about the safety of intelligent packaging. Extensive public education and promotion of such systems is needed. Finally, the use of intelligent packaging can give a significant advantage in the market due to the possibility of the real-time control of food products. If all steps are taken to bring such solutions to the market, intelligent packaging will help producers and consumers in various spheres.

Hence, it is important to continue research into intelligent packaging given the multitude of benefits it can offer.

## Figures and Tables

**Table 1:** Classification of intelligent packaging according to the operation mode [9].

Intelligent Packaging		
Component	Base	
**Sensors**	Gas sensors	Bio sensors
	Fluorescence based Oxygen Sensors	
**Indicators**	Microbial growth & freshness indicators	Time temperature indicators
	Gas leakage/concentration indicators	
**Tags/Barcodes**	1-D barcode	2-D barcode
	QR code	RFID tags
**Holograms**		
**Thermochromic inks**		

## Data Availability

Not applicable.
